# Evaluation of the effects of methadone and tramadol on postoperative analgesia and serum interleukin-6 in dogs undergoing orthopaedic surgery

**DOI:** 10.1186/s12917-014-0194-7

**Published:** 2014-09-06

**Authors:** Larissa B Cardozo, Lourenço C Cotes, Marcia A P Kahvegian, Maria Fernanda C I Rizzo, Denise A Otsuki, Cassio R A Ferrigno, Denise T Fantoni

**Affiliations:** Program of Postgraduate Anesthesiology, Faculdade de Medicina, Universidade de São Paulo, São Paulo, Brazil; Department of Surgery (VCI), Faculdade de Medicina Veterinária e Zootecnia, Universidade de São Paulo, São Paulo, Brazil; Discipline of Surgery and Anesthesiology, Universidade Cruzeiro do Sul, São Paulo, Brazil; Laboratory of Medical Investigation, LIM08/Anestesiologia, Faculdade de Medicina, Universidade de São Paulo, São Paulo, Brazil

## Abstract

**Background:**

Acute postsurgical pain is of great interest due to potential risk of becoming chronic if not treated properly, worsening patient’s recovery and quality of life. Twenty-eight dogs with ruptured cruciate ligaments were divided into three groups that received intramuscular injections of 4 mg/kg of tramadol (TRA), 0.5 mg/kg of methadone (MET0.5), or 0.7 mg/kg of methadone (MET0.7). Physiological parameters (heart and respiratory rates and blood pressure) were evaluated at specified times: baseline (TBL), 1 (T1), 2 (T2), 4 (T4), 6 (T6), and 24 (T24) hours after premedication. Pain scores were described by visual analogue scale (VAS), modified Glasgow Composite, and Colorado University Acute Pain scales. Blood samples for measurement of interleukin (IL)-6 were collected at TBL, T1, T6, and T24. This was a prospective, randomised investigation to evaluate the efficacy of tramadol and methadone as premedications in dogs undergoing osteotomies.

**Results:**

There were no statistically significant differences between groups with respect to age, weight, gender, surgery time, and time to extubation. Heart rate, respiratory rate, and blood pressure values were maintained within acceptable ranges, and a reduction was observed at T2 in MET0.5 and MET0.7 compared with TBL. Increases in VAS scores were observed in TRA at T4 compared with TBL, T1, and T24 and between T1 and T6 (p < 0.001). In MET0.5, there was significant increase in VAS score at T4 compared with T1 (p < 0.001). TRA and MET0.5 showed significantly higher mean ± SD VAS scores (3.4 ± 2.5 and 2.5 ± 2.6, respectively) than MET0.7 (1.1 ± 1.5) at T4 (p < 0.001). TRA showed greater demand of rescue analgesia (four animals in T4 and two in T6) (p < 0.037). There were no statistically significant differences in sedation scores, Colorado Scale scores, or interleukin levels between groups and time points.

**Conclusions:**

Methadone given as premedication in doses of 0.7 mg/kg was better at controlling pain compared with lower doses and tramadol. However, dosage increases, administered as rescue analgesia, promoted adequate pain control even in tramadol group. Influence of these analgesics on IL-6 release could not be demonstrated, but significant levels were not found.

**Electronic supplementary material:**

The online version of this article (doi:10.1186/s12917-014-0194-7) contains supplementary material, which is available to authorized users.

## Background

Postoperative pain management is a major concern and influences recovery time, quality of life, and surgical outcome [1,2]. Acute pain alters metabolic and hemodynamic systems, facilitates the release of the inflammatory cascade, and triggers a sympathetic stress response [3]. Serum values of cortisol, glucose, and interleukins may provide important information, in association with clinical parameters and pain scales, in the study of different pain conditions and proposed treatments in dogs [4]. Pro-inflammatory cytokines, such as interleukin-6 (IL-6), show a high association with acute pain because they sensitise the nervous system. IL-6 levels in plasma are detectable 60 minutes after injury, peaking at 4-6 hours, and are proportional to tissue injury [5-9].

An adequate approach to postoperative pain control is preventive analgesia. It has been suggested that the administration of non-steroidal anti-inflammatory drugs (NSAIDs), opioids, or local anaesthetics prior to injury reduces central sensitisation, analgesic consumption, and the use of rescue analgesia [10,11]. Opioids are commonly used for sedation and the treatment of postoperative pain in small animals [4,12-14]. Although it is well known that they may cause immune suppression in humans by helping to control the release of inflammatory substances [15-17], there is little information regarding opioid action on the canine immune system.

Methadone is an opioid that has recently been introduced into routine veterinary medicine practice, and its analgesic effects have been of great interest. It acts as a Mu-opioid receptor agonist and as an N-methyl-D-aspartate (NMDA) receptor antagonist [18,19]. Additionally, methadone is considered to be as powerful as morphine but causes fewer side effects, such as nausea, vomiting, and dysphoria [20]. Tramadol is a well-known synthetic opioid with low Mu-opioid agonist affinity (6000 times less than morphine), and its efficacy is due to two different mechanisms, namely, it interacts with Mu-opioid receptors and has effects on serotonin and norepinephrine reuptake. O-desmethyl tramadol (M1), a product of tramadol hepatic metabolism, is known to present a higher affinity for Mu receptors [21,22]. It is also believed to have an immunostimulatory effect, as it decreased IL-6 levels in a model of incisional pain in rats [17].

The aim of the present study was to evaluate the effects of methadone and tramadol on postoperative pain in dogs undergoing tibial plateau levelling osteotomy (TPLO) and their influence on serum IL-6 levels.

## Methods

### Animals

Twenty-eight client-owned dogs admitted to the Orthopedics and Trauma Laboratory that presented with cranial cruciate ligament ruptures were included in the study. All the animals were judged as healthy based on physical examinations, complete blood cell counts, and serum biochemical analyses before surgery. Animals presenting with any alterations in these parameters were excluded from the study, as were those given any type of analgesic drug prior to the study. Owners of the eligible dogs agreed to their inclusion in the study by signing a consent agreement form. Food and water were withheld for 12 hours prior to surgery.

### Experimental design

A complete physical evaluation was performed for all animals before any other intervention. Pain scores were assessed by several scales (the visual analogue scale, the modified Glasgow Composite Measure Pain Scale (CMPS), and the Colorado University Canine Acute Pain Scale) at baseline for later comparison with the postoperative period. Blood samples were collected for the assessment of IL-6 levels using a specific kit for canine serum (Quantikine®, R&D Systems, Minnesota, USA) before premedication (baseline (TBL)) and after surgery (1, 6, and 24 hours after premedication). Animals were randomly assigned to three different groups as follows: the TRA group (n = 9) received 4 mg/kg of tramadol (Tramadon®, Cristália, São Paulo, Brazil); the MET0.5 group (n = 10) received 0.5 mg/kg of methadone (Mytedon®, Cristália, São Paulo, Brazil); and the MET0.7 group (n = 9) received 0.7 mg/kg of methadone. All drugs were injected intramuscularly as premedication. The study was approved by the Ethical Committee of Faculdade de Medicina (0169/05) and Faculdade de Medicina Veterinária e Zootecnia (1077/2007) from the Universidade de São Paulo.

### Procedure

Sixty minutes after premedication, the cephalic vein was catheterised for fluid administration (lactated Ringer’s solution, Baxter Hospitalar, SP, Brazil) at a rate of 10 mL/kg/h and for drug infusions. Anaesthesia was induced with IV propofol (5-7 mg/kg) (Propovan®, Cristália, São Paulo, Brazil) to achieve the desired effects (i.e., muscular relaxation and loss of protective reflexes), and intubation was then performed with an adequately sized endotracheal tube.

General anaesthesia was maintained with isoflurane (Isoflorane®, Cristália, São Paulo, Brazil) in 100% oxygen under positive pressure ventilation (10 cmH_2_O) in a circle rebreathing system, with the respiratory rate adjusted to maintain the end-tidal concentration of carbon dioxide between 35 and 45 mmHg. Heart and respiratory rates, direct arterial blood pressure, and oxygen saturation (assessed by pulse oximetry) were monitored by a multiparameter monitor (DX 2020, Dixtal, São Paulo, Brazil). A 20-gauge catheter was aseptically placed on the dorsal pedal artery and connected to the monitor’s pressure transducer with zero reference pressure at the level of the heart. End-tidal concentrations of isoflurane (EtISO) and carbon dioxide (EtCO_2_) were monitored by a gas analyser and capnograph (Poet® IQ, Criticare Systems Inc., Waukesha, USA), with the EtISO initially set at 1.4%. The gas analyser was calibrated before each experiment with a standard gas mixture (White Martins Gases Especiais, São Paulo, Brazil).

In the case of hypotension (MAP <65 mmHg), ephedrine (0.1 mg/kg) was administered. One bolus of fentanyl (3 μcg/kg) was administered intravenously just before starting the osteotomy. Whenever there was a change in heart rate or blood pressure greater than 20% compared with pre-stimulation values, a 0.1% increase in isoflurane was administered. Temperature was maintained between 37 and 38°C with the use of a warming blanket (Gaymar T-pump TP500, Kent Scientific Corporation, Connecticut, USA). An 18-gauge catheter was aseptically placed in a jugular vein to facilitate subsequent blood sample collections. All surgeries were performed by the same experienced surgeon (senior of the Orthopedics and Traumatology Department).

Sedation scores were assessed 30 minutes after premedication injection and at 1 and 3 hours after surgery. A scale proposed by Valverde et al. [23], ranging from 0 to 3, was used for this purpose, with 0 = no effect, 1 = mild effect (animal less alert but still active), 2 = moderate effect (drowsy and recumbent but can walk), and 3 = severe effect (very drowsy and unable to walk).

Physiological parameters were recorded at 1 (T1), 2 (T2), 4 (T4), 6 (T6), and 24 (T24) hours after premedication. Pain was assessed at the same time points after analgesic injection, except at T2, using the VAS and modified Glasgow Composite Measure Pain Scale [24,25], each of which ranged from 0 to 10, where 0 indicates no pain and 10 indicates the worst pain possible. The Colorado University Canine Acute Pain Scale was also used and ranged from 0 to 4, with 0 indicating minimal pain and 4 indicating severe pain (see Additional file 1). All parameters were assessed by the same observer (LBC), who was unaware of the analgesic treatment and has experience in pain evaluation.

If scores for the VAS, Colorado, and Glasgow scales were higher than 4, 2, and 4, respectively, rescue analgesia was administered as follows to reduce the influence of other analgesic drugs on pain and IL-6 levels: 1 mg/kg of tramadol for the TRA group and 0.2 mg/kg of methadone for the MET0.5 and MET0.7 groups. Animals were re-evaluated 20 minutes after the administration of rescue medication, and if signs of pain persisted, 0.2 mg/kg of morphine was administered and the animal was withdrawn from the study. The animals were observed after surgery, sent home, and then returned 24 hours later for evaluation.

The occurrence of any adverse effects, such as nausea, vomiting, dysphoria, mydriasis, or intense salivation, was recorded, as well as the number of animals requiring rescue analgesia. NSAIDs were not administered during the evaluation period, but carprofen (2.2 mg/kg twice a day, orally) was prescribed for 10 days postoperatively, as is usually recommended by surgeons. Blood collection was performed at TBL, T1, T6, and T24 for measurement of serum IL-6 concentrations using a commercial ELISA kit with canine-specific monoclonal antibodies. Blood collection, centrifugation to obtain sera, and laboratory techniques for IL-6 measurement were performed as previously described by Martins et al. [4].

### Statistical analysis

Data were analysed with the Kolmogorov-Smirnov test for normally distributed data using the software package Sigma Plot v11.0 (Systat Software, Chicago, USA). Physiological parameters were analysed by analysis of variance (ANOVA) for repeated measurements, followed by the Tukey test. Pain and sedation scores were compared at different times by Friedman’s test, followed by the Tukey test. Groups were compared by the non-parametric Kruskal-Wallis test, followed by a post-hoc Dunn’s test. A significance level of p < 0.05 was used, and values are presented as the mean ± SD.

## Results

The animals ranged in age from 1 to 7 years, with a mean weight (±SD) of 33.0 (±11.2) kg. The mean surgery time and time to extubation did not differ significantly between groups (Table 1). A significant difference was found in heart rate (HR), respiratory rate (RR), and diastolic blood pressure (DBP) 2 hours after premedication administration in all groups compared with baseline values (Table 2). This result occurred because this time point was evaluated during surgery when all animals were under general anaesthesia with a volatile anaesthetic agent (isoflurane).

**Table 1:** **Mean surgery and extubation times by treatment group**

**Group**	Surgery time* (mean ± SD)	Extubation time* (mean ± SD)
**TRA**	95.3 ± 17.4	6.6 ± 2.4
**MET 0.5**	98.2 ± 15.2	7.2 ± 1.6
**MET 0.7**	98.5 ± 12.8	7.3 ± 2.2

*measured in minutes.

**Table 2:** **Phisyological parameters measure during time points by treatment group** ^**#**^

		**Time TBL**	**point T1**	**T2**	**T4**	**T6**	**T24**
**HR**	**TRA**	136 ± 25	123 ± 12	101 ± 25^b^	121 ± 25^b^	143 ± 25^*,a,b^	151 ± 27^a^
	**MET 0.5**	133 ± 25	107 ± 30^*^	83 ± 20^*^	110 ± 26^b^	106 ± 27^*^	121 ± 8
	**MET 0.7**	140 ± 23	117 ± 16	64 ± 17^*^	86 ± 26^*^	105 ± 17^*^	140 ± 14
**RR**	**TRA**	56 ± 9	46 ± 11^*^	10 ± 2^*^	26 ± 13^*^	36 ± 18^*^	55 ± 10
	**MET 0.5**	52 ± 13	55 ± 15^*^	10 ± 2^*^	31 ± 21^*^	41 ± 17^*^	60 ± 10
	**MET 0.7**	58 ± 7	60 ± 5^*^	10 ± 2^*^	25 ± 14^*^	39 ± 14^*^	52 ± 18
**SAP**	**TRA**	183 ± 15	161 ± 17^b^	106 ± 17^*^	156 ± 25^*^	163 ± 24	176 ± 17^a^
	**MET 0.5**	168 ± 24	146 ± 43^b^	90 ± 17^*^	147 ± 19	147 ± 23	173 ± 10
	**Met 0.7**	179 ± 23	187 ± 21	98 ± 20^*^	120 ± 34	170 ± 23	165 ± 40
**MAP**	**TRA**	121 ± 3	128 ± 18	89 ± 19^*^	104 ± 28	119 ± 25	140 ± 26
	**MET 0.5**	126 ± 19	109 ± 31^b^	77 ± 13^*^	108 ± 23	108 ± 23	123 ± 9
	**MET 0.7**	134 ± 24	137 ± 18	74 ± 14^*^	110 ± 21^*^	124 ± 21	122 ± 14
**DAP**	**TRA**	91 ± 5	111 ± 5^a^	78 ± 17	87 ± 26	92 ± 23	100 ± 5^a^
	**MET 0.5**	100 ± 19	85 ± 28^b^	60 ± 12	60 ± 18	87 ± 23	77 ± 12^b^
	**MET 0.7**	106 ± 22	107 ± 18	58 ± 14	84 ± 16	96 ± 21	100 ± 18

^#^ *HR*: Heart rate in beats per minute; *RR*: Respiratory rate in breaths per minute; *SAP, MAP* and *DAP*: Systolic, Mean and Diastolic arterial pressures in mmHg.

*:differs from TBL; a: differs from MET 0.5, b: differs from MET 0.7; p < 0.05.

None of the animals required ephedrine treatment for hypotension. No values were lower than those considered normal under these conditions, except for the value in one animal in the MET0.7 group that had a HR ≤ 60 beats per minute (37 bpm) and required an intravenous atropine sulphate injection (0.025 mg/kg). Groups that received methadone treatment had lower mean HR values compared with those in the tramadol treatment group, although these differences were not significant. The mean RR values decreased after premedication compared with those at baseline in all groups (p < 0.001).

**Figure 1:**
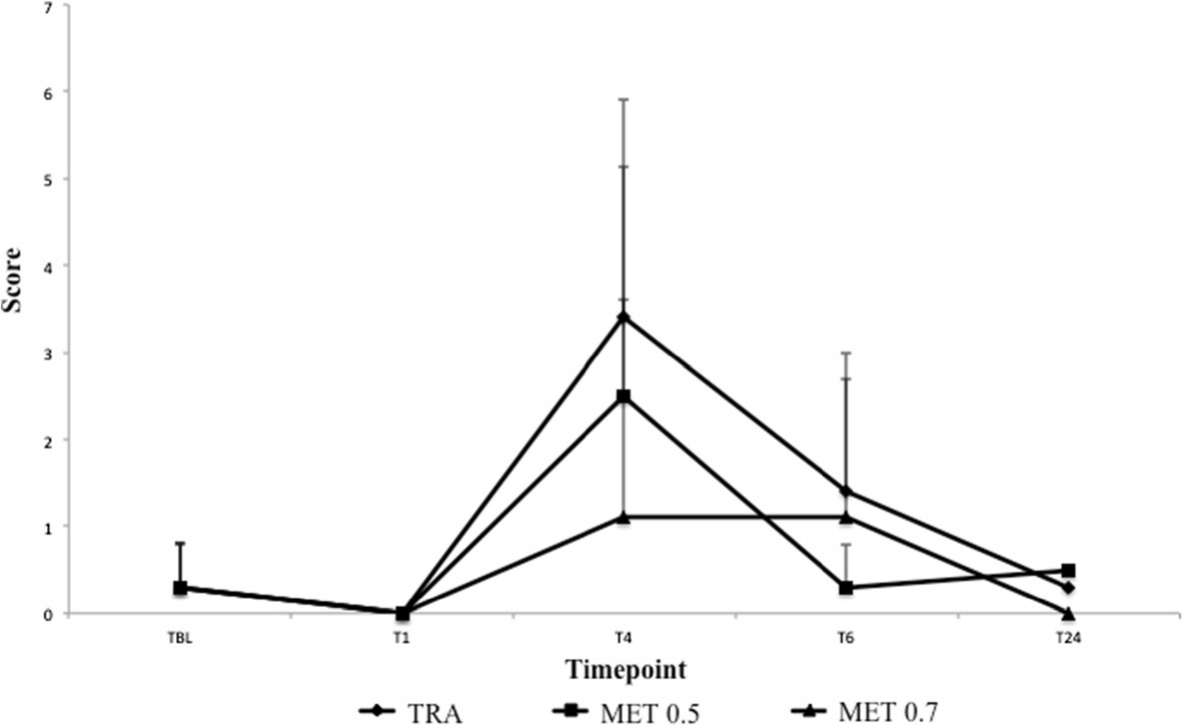
**Mean ± SD VAS scores measured at timepoints by treatment groups.**

Sedation scores did not differ between groups or time points, with mean values lower than 2 at every evaluation (Table 3). There were no significant differences between groups in the Colorado Pain Scale scores, and there were no mean values greater than 2 (Table 4). In the TRA group, VAS scores were increased at T4 compared with those at TBL, T1, and T24 (p < 0.001). In the MET0.5 group, there was a significant increase at T4 compared with at T1 (p < 0.001). No significant difference was found within the MET0.7 group, which presented the lowest mean VAS scores at T4 (1.1 ± 1.5), while the mean scores in the TRA and MET0.5 groups were 3.4 ± 2.5 and 2.5 ± 2.6, respectively (p = 0.049) (Figure 1).

**Table 3:** Sedation scores* by treatmetn group - median (upper-lower value)

**Group**	**TBL**	**T1**	**T4**	**T6**	**T24**
**TRA**	0 (0-0)	1 (0-1)	1 (0-1)	0 (0-1)	0 (0-0)
**MET 0.5**	0 (0-0)	1 (0-2)	1 (1-2)	1 (0-1)	0 (0-0)
**MET 0.7**	0 (0-0)	2 (1-2)	2 (1-2)	1 (0-2)	0 (0-0)

*according to Valverde et al. [23].

**Table 4:** **Colorado University pain scale scores by treatment group - median (upper-lower value)**

**Group**	**TBL**	**T1**	**T4**	**T6**	**T24**
**TRA**	0 (0-0)	0 (0-0)	1.5 (0-3)	1 (0-2)	0 (0-1)
**MET 0.5**	0 (0-0)	0 (0-0)	1 (1-3)	0 (0-0)	0 (0-1)
**MET 0.7**	0 (0-0)	0 (0-0)	0.5 (0-2)	0 (0-1)	0 (0-0)

Dogs in the MET0.7 group also had lower scores on the modified GCPS (2.2 ± 1.55), indicating better pain control compared with that provided by the other analgesic treatments (3.39 ± 2.78 in the TRA group and 3.08 ± 2.43 in the MET0.5 group) at T4 (p < 0.001). Six animals in the TRA group required rescue analgesia (four at T4 and two at T6), as did one animal in the MET0.5 group at T4. Pain control was found to be adequate thereafter, with no animal requiring further medication (i.e., morphine).

Treatment groups did not differ significantly with regard to serum IL-6 levels. An increase was observed in all groups compared with baseline values, although this increase was not significant.

## Discussion

This study evaluated and compared the effects of methadone and tramadol on postoperative pain and IL-6 levels in dogs undergoing orthopaedic surgery. VAS and modified Glasgow Composite Measure Pain Scale scores showed that methadone yielded better pain control. Analgesia improved with an increased dosage increment, suggesting that methadone-induced analgesia is dose-dependent. Nevertheless, all treatments had effects on IL-6 by controlling its systemic release after surgery.

Adequate pain control is a major concern in the perioperative period. Acute pain may delay recovery and wound healing, reduce mobilisation and food and water consumption, and evolve to chronic postsurgical pain if not treated properly. It is well known that opioid administration prior to surgery reduces postoperative pain scores and analgesic drug requirements. Methadone was recently introduced into routine hospital use as a treatment for heroin addicts. However, it has been shown that it may also have significant effects on acute, chronic, oncologic, and neuropathic pain, making it an excellent alternative to the use of morphine, as methadone causes fewer side effects and has a multi-action profile, acting as a Mu-opioid receptor agonist and an NMDA receptor antagonist [20,26,27].

A control (no analgesic treatment) group was not included in this study because it is unethical to perform orthopaedic surgery that has the possibly of causing a high degree of pain without analgesic administration. The authors are aware that the absence of a control group may decrease the confidence in the results. Tramadol was chosen as a positive control for methadone because it is a well-known drug for acute pain control in small animals, providing the same analgesic effect as morphine in equipotent doses, and because of its immunomodulatory properties [12,13,21,28].

Animals in the groups treated with methadone exhibited higher sedation scores than those treated with tramadol, in accordance with previous data [13]. In this study, tranquilisers were not administered as part of the premedication regimen due to the aim of achieving a lighter degree of sedation to reduce interference with the pain evaluation. Although sedation scores were higher in the methadone groups, no significant differences in these scores were observed. Methadone is claimed to promote a more intense degree of sedation than tramadol. The degree of sedation resulting from tramadol administration is less intense, even when acepromazine is co-administered [13], but this result has already been described in dogs [29-31]. However, isolated use of opioids is recommended, especially in animals that can be easily handled, because the degree of sedation in this study did not exceed 1 (mild) on the sedation scale.

Respiratory depression may be induced by the use of opioids, depending on the dosage and administration route [13,32]. Importantly, this effect was not observed in this study, which may be related to the intramuscular administration employed, which alters absorption kinetics and leads to fewer side effects, representing an advantage over morphine.

Dysphoria was not observed in any of the groups. This is a very interesting finding because dysphoria is a common side effect of opioids in dogs and also an effect observed in other species after tramadol administration [33-35]. This result may be related to the dosage employed in this study or the presence of injury/pain. In pain-free conscious dogs, Garofalo et al. [32] reported the presence of dysphoria in all animals after 1 mg/kg of methadone was intravenously administered. In previous studies where tramadol was used for the control of postoperative pain after ovariohysterectomy, mandibulectomy, and maxillectomy, dysphoria was not observed [4,12,28].

No other adverse effects were observed in any of the treatment groups during the observation period. However, eight animals in the TRA group exhibited intense salivation after surgery, possibly due to nausea [36]. Postoperative nausea and vomiting are a major concern associated with human anaesthesia and are described to be as limiting as pain by the patients [37]. These symptoms are described in humans after the intravenous injection of tramadol and are related to prolonged administration. None of the animals in this study presented any other side effects during the observation period.

The absence of vomiting after tramadol and methadone administration in the pre-anaesthetic period is considered, in our view, an important advantage of these agents. Morphine, oxymorphone, and hydromorphone are frequently utilised as premedications in dogs and cause a high incidence of vomiting [23,38], which may lead to aspiration pneumonia [38,39]. In humans, vomiting is considered one of the main anaesthetic complications and is associated with a high incidence of intubation and acute respiratory distress syndrome (ARDS) [37]. In veterinary medicine, there are few published reports of this post-anaesthetic complication, although it has recently been shown to have a much higher incidence in animals (0.17%) than in humans (0.014 – 0.05%) [39].

Regarding pain assessment, animals received rescue analgesia if any of the pain scale scores were increased. The scores for all animals returned to baseline levels after opioid dose adjustment (as cited in the Methods section), demonstrating that the effects of opioid analgesia were dose-dependent under the study conditions. There was a slight correlation between the three pain scales. The modified Glasgow Composite Measure Pain Scale showed a higher association with patients’ clinical statuses. This result most likely occurred because this scale allows for a more detailed assessment of behaviours, making it suitable as a measure of pain in clinical situations [25].

Acute injuries, such as surgery, have been reported to cause an early response with cytokine production. However, this response in not related to the duration of surgery and is localised to the site of injury. IL-6 levels show a high association with pain and are related to pain intensity [40]. A recent study evaluating IL-6 levels in dogs with oral cancer revealed a baseline IL-6 value of 100 pg/ml and a mean peak level of 140 pg/ml after surgery during the observation period. In other studies comparing the IL-6 levels of healthy and leishmania-positive dogs, the authors found a higher value of 16.2 pg/ml in infected dogs [4,41]. In the present study, there was a similar increase in IL-6 levels in all treatment groups, representing an immune response to surgical stimulation, with mean values under 185 pg/ml. These findings are in accordance with studies evaluating IL-6 levels in dogs undergoing laparoscopic surgery, in which values increased above baseline 2 to 6 hours after surgery, with mean values not exceeding 100 pg/ml [42].

## Conclusions

In conclusion, methadone administered as premedication at a dose of 0.7 mg/kg had a better effect on pain control after orthopaedic surgery compared with a lower dosage of the same drug and with tramadol. However, the dosage increases, even tramadol increases, administered as rescue analgesia promoted adequate pain control. The influence of the two analgesics on IL-6 release could not be demonstrated in the present data, but significantly high levels of IL-6 were not found.

## Additional file

Additional file 1:
**Colorado State University Canine Acute Pain Scale.**
