# Supplementary material for: Evaluation of the effects of methadone and tramadol on postoperative analgesia and serum interleukin-6 in dogs undergoing orthopaedic surgery
Source: BMC Vet Res. 2014 Sep 6;10:194. doi: 10.1186/s12917-014-0194-7 (PMC4173003; doi:10.1186/s12917-014-0194-7)
Supplement: Additional file 1: — Colorado State University Canine Acute Pain Scale. [file 12917_2014_194_MOESM1_ESM.pdf]

Date ______________________

Time ______________________

Colorado State University
Veterinary Medical Center

Canine Acute Pain Scale

Rescore when awake
- ☐ **Animal is sleeping, but can be aroused - Not evaluated for pain**
- ☐ **Animal can't be aroused, check vital signs, assess therapy**

Pain Score	Example	Psychological & Behavioral	Response to Palpation	Body Tension
0	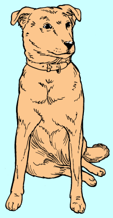	☐ **Comfortable** when resting<br>☐ **Happy, content**<br>☐ Not bothering wound or surgery site<br>☐ Interested in or curious about surroundings	☐ **Nontender** to palpation of wound or surgery site, or to palpation elsewhere	Minimal
1	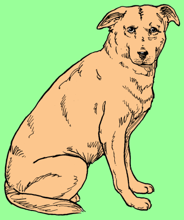	☐ **Content to slightly unsettled** or restless<br>☐ **Distracted easily** by surroundings	☐ **Reacts to palpation** of wound, surgery site, or other body part by **looking around, flinching,** or **whimpering**	Mild
2	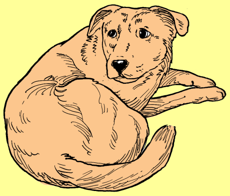	☐ Looks **uncomfortable** when resting<br>☐ May **whimper** or cry and may **lick or rub wound** or surgery site when unattended<br>☐ Droopy ears, **worried facial expression** (arched eye brows, darting eyes)<br>☐ **Reluctant to respond** when beckoned<br>☐ **Not eager to interact** with people or surroundings but will look around to see what is going on	☐ Flinches, whimpers cries, or guards/pulls away	Mild to Moderate<br>**Reassess analgesic plan**
3	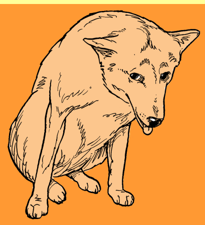	☐ **Unsettled, crying, groaning, biting or chewing** wound when unattended<br>☐ **Guards or protects** wound or surgery site by altering weight distribution (i.e., limping, shifting body position)<br>☐ **May be unwilling to move** all or part of body	☐ May be **subtle** (shifting eyes or increased respiratory rate) if dog is too painful to move or is stoic<br>☐ May be **dramatic**, such as a sharp cry, growl, bite or bite threat, and/or pulling away	Moderate<br>**Reassess analgesic plan**
4	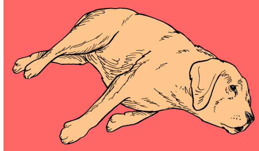	☐ **Constantly groaning or screaming** when unattended<br>☐ May bite or chew at wound, but unlikely to move<br>☐ **Potentially unresponsive** to surroundings<br>☐ **Difficult to distract** from pain	☐ **Cries at non-painful palpation** (may be experiencing allodynia, wind-up, or fearful that pain could be made worse)<br>☐ May react aggressively to palpation	Moderate to Severe<br>**May be rigid to avoid painful movement**<br>**Reassess analgesic plan**

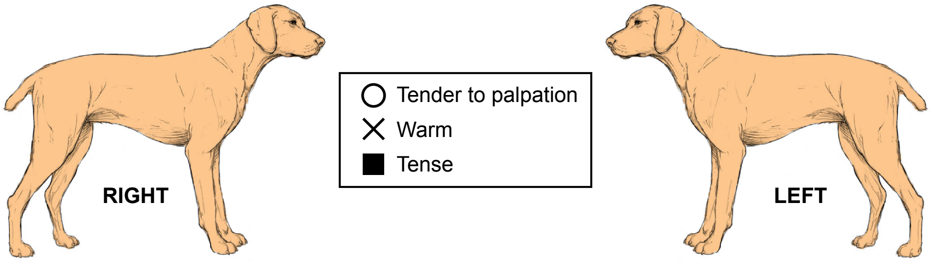

Comments ____

© 2006/PW Hellyer, SR Uhrig, NG Robinson

Supported by an Unrestricted Educational Grant from Pfizer Animal Health
